# Memantine Usage for Management of Korsakoff Syndrome in the Setting of Chronic Alcohol Use and Unspecified Eating Disorder

**DOI:** 10.7759/cureus.13846

**Published:** 2021-03-12

**Authors:** Varun Reddy, Andrea Hernandez, Leah Grossman, Debra Angelo, Johnathan Frunzi

**Affiliations:** 1 Internal Medicine, Medical Center of Trinity, Trinity, USA

**Keywords:** wernicke-korsakoff syndrome, memantine, nmda receptor antagonist, quetapine

## Abstract

Korsakoff syndrome is a neuropsychiatric condition frequently seen as a progression of Wernicke’s encephalopathy and is often associated with long-term alcohol abuse. It is characterized by further cognitive impairments, such as indiscriminate anterograde and retrograde amnesia, in addition to executive function deficits. As the syndrome is a result of severe thiamine deficiency, its management primarily focuses on nutritional replenishment and electrolyte maintenance. In recent years, a few published reports have detailed the off-label use of Food and Drug Administration (FDA) approved drugs for Alzheimer’s in an attempt to treat neurocognitive deficits associated with Korsakoff patients. In this particular case, we note subjective improvement in cognition after initiating memantine, an N-Methyl-d-aspartate (NMDA) receptor antagonist.

## Introduction

Wernicke-Korsakoff syndrome is a spectrum of neuropsychiatric deficiencies that result from severe thiamine deficiency. Wernicke’s encephalopathy classically presents with a sequelae of ophthalmoplegia, ataxia, and altered mental status, and is typically acute on presentation but reversible. Korsakoff syndrome (KS) has a similar yet higher acuity of neurocognitive deficits, generally appears after an initial episode of Wernicke’s and is considered chronic, progressive, and irreversible, due to direct damage to neurons. These deficits will often present as difficulty learning, poor anterograde memory with confabulations, and difficulty with planning or problem-solving [1]. Any condition that causes prolonged thiamine deficiency can lead to Korsakoff syndrome, which is often a clinical diagnosis. Chronic alcohol abuse is the most common cause, however, eating disorders and chemotherapy are also potential contributory factors [2]. Thiamine is essential to brain cell metabolism, and prolonged deficiency can cause glutamate to accumulate in peripheral tissue, leading to excitotoxicity and localized inflammation [3]. The result of this is neuronal damage, gliosis and edema primarily in the thalamus, mammillary bodies, and corpus callosus. MRI findings of increased T2-attenuation in these areas are considered pathognomonic, however are seen in only a fraction of diagnosed cases [4]. Management consists primarily of thiamine repletion and electrolyte management as a means of preventing further damage. No secondary medications, including nootropic or memory-enhancing medications, are FDA-approved for treatment.

Alzheimer’s disease is a neurodegenerative disorder that can present with some similar cognitive impairments. It is primarily characterized by a progressive decline in multiple cognitive metrics ultimately leading to interference in the ability to perform basic activities of daily living (ADL). Alzheimer’s disease is suspected to be a result of accumulation of amyloid β plaques in the brain. These plaques interfere with synaptic signaling and can trigger microglia recruitment to the area [5]. Microglial activation and the subsequent local inflammatory response lead to more permanent neuronal damage [6]. Another potential mechanism of neurodegeneration is the accumulation of glutamate. Glutamate is an excitatory neurotransmitter that at high levels can trigger cell death [7]. Currently, treatment options for afflicted patients are limited. Acetylcholinesterase inhibitors such as donepezil, or N-Methyl-d-aspartate (NMDA) antagonist/dopamine antagonists like memantine, are the only FDA-approved options in the United States. These medications are thought to work by compensating for the loss of cholinergic neurons or by preventing accumulation of toxic levels of glutamate, respectively.

As both Korsakoff syndrome and Alzheimer’s disease can present with some similar cognitive changes, namely anterograde amnesia, it has been proposed that medications used for Alzheimer’s could elicit similar improvement in Korsakoff syndrome. To date, there have been very limited off-label trials of these nootropic medications in KS patients, leaving the question of clinical use and efficacy in Korsakoff syndrome unanswered. In this case, we observe a patient diagnosed with Korsakoff syndrome and her response to memantine.

## Case presentation

A 47-year-old Caucasian female with a past medical history of major depressive disorder, chronic alcohol abuse, and an unspecified eating disorder was brought to the hospital by emergency medical services under the Florida Mental Health Act due to new-onset altered mental status. The patient had been found to be too weak to stand and her only reported oral intake had been alcohol with no food for an undefinable amount of time. Examination in the emergency room revealed a tangential thought process and poor responses to questioning. She believed she was in her home and the hospital staff were friends and family. She was found to be tachycardic with a heart rate of 131 beats per minute (bpm), blood pressure 105/75 mm Hg, respirations 16 breaths per minute with a 99% pulse oxygen level, and afebrile 37.3⁰C (99.1⁰F). On physical exam, the patient was noted to be cachexic and tremulous with a body mass index (BMI) of 15.9kg/m2. Horizontal nystagmus was noted. The rest of the physical exam was unremarkable. Laboratory analysis abnormalities showed leukocytosis 19.04 x 103/uL, hypokalemia with potassium 3.2 mmol/L, slight hypernatremia 146 mmol/L, creatinine 2.2 mg/dL, blood urea nitrogen (BUN) 59 mg/dL, glucose 144 mg/dL, and mild hypercalcemia 10.5mmol/L. Liver function was normal as well as albumin levels. Urine toxicology was negative and alcohol level was < 3 mg/dL. Patient met criteria for sepsis as she was tachycardic with acute kidney injury with elevated creatinine, leukocytosis, and an elevated lactic acid level of 4.97 mmol/L (normal 0.90-1.70 mmol/L) with altered mental status. Lipase levels were found to be elevated at 1234 unit/L. Chest x-ray and computed tomography (CT) head were unremarkable. She was admitted to the intensive care unit (ICU) with severe sepsis, altered mental status, acute kidney injury, and failure to thrive.

The patient was started on high-dose thiamine supplementation with close monitoring of electrolytes. She experienced persistent encephalopathy, ataxia, and nystagmus. The patient was retaining urine on bladder scan and a Foley catheter was placed with a urine sample sent for urinalysis. She was started on intravenous ceftriaxone. Magnetic resonance imaging (MRI) of the brain without contrast also showed no acute intracranial processes. The initial urinalysis returned positive and urine cultures were sent out. The urine culture was positive for *Escherichia coli* and antibiotics were adjusted according to the sensitivity panel. The patient did not show any improvement in mental status following completion of the antibiotic course. The patient continued to display fluctuating mental status, believing she was at home with her parents and, on rare occasions, understood that she was in the hospital. The patient also showed increased agitation in the late evenings where she was found to be combative and tearful. These episodes required extra nursing support with frequent reorientation. After discussion with Neurology and Psychiatry, it was determined the patient’s cognitive status was likely permanent and was classified as Korsakoff syndrome. As the primary team, multiple antipsychotics were attempted to stabilize her moods and agitation, including varying doses of haloperidol, ziprasidone, and quetiapine. Eventually we settled on a scheduled low dose of quetiapine, which showed only minimal improvement in patient’s mental status.

By this point, the patient had been hospitalized for over two months. There were multiple barriers making a safe discharge plan difficult to establish. We conversed with the patient’s sister and power of attorney (POA) regarding possible alternative treatments, including initiation of memantine. After discussing the risks and benefits, we agreed to initiate memantine. Notably, the patient’s sister reported a family history of alcoholism in both parents and a prodrome of altered mental status for a month-long period prior to their father’s death. The patient’s mother’s cause of death was unknown. The patient and her sister had last spoken one month prior to admission and her sister reported the patient was alert and oriented x4 at that time with no prior history of Wernicke’s or memory deficits.

A Folstein mini-mental state exam (MMSE) was first performed prior to initiation of the medication on which the patient scored an 18/30. Her primary deficits were in cognition and recall. She showed no deficits in attention, calculation, or language skills. She was started on a low dose of memantine 5 mg twice a day and observed. After one week of treatment, she appeared to have more frequent, intermittent episodes of clarity during which she was oriented to place and was able to recall she was in the hospital, however, she could not recall reasons for hospitalizations despite daily reorienting. She was consistently disoriented to time and self. Long-term memory including details regarding childhood and early adult life remained preserved. Nursing staff noticed she appeared to be calmer in the evenings and her mood appeared less labile. Her MMSE after one week of treatment with memantine was 19/30. During the remainder of her admission, we avoided making any changes to her quetiapine. Her memantine was increased to a maintenance dose of 10 mg twice a day with continued observation of her cognitive function and weekly MMSEs. Her mental status continuously fluctuated over the next few weeks, ranging between 18-22/30. After 119 days, she was deemed medically optimized for discharge to a long-term memory care facility.

## Discussion

In this case, we observed a patient with history of chronic alcohol abuse combined with an eating disorder who went on to develop Korsakoff syndrome as a result of severe prolonged thiamine deficiency. In this particular case, the diagnosis of Korsakoff syndrome was made clinically, based on clinical findings and a history of 20+ years of alcohol abuse mixed with an eating disorder. There were no objective findings in her MRI consistent with Korsakoff syndrome. Per Zuccoli et al., MRI findings for patients with Korsakoff syndrome can vary based on whether the inciting factor is alcoholic or nonalcoholic in nature [8]. The mixed nature of our patient’s history may point to why her MRI was negative, however it is impossible to rule out the existence of another underlying disorder that may have contributed to her overall cognitive decline.

Another major aspect of this report is the MMSE. Though limited, it was able to serve as an objective means through which we could measure cognitive changes. Throughout her extended hospitalization, we observed fluctuations in her cognition. Through use of the Folstein Mini-Mental State examination, we identified that the patient had no speech or problem-solving deficits. She did have significant difficulty with self-orientation and with short-term recall. We also observed intense mood swings with sundowning. She had been started on thiamine supplementation early in her admission, with no significant improvement in status. After some consideration and discussion with family, we started the patient on memantine. This medication was chosen over donepezil as the patient was already on quetiapine and we wanted to minimize the risk of cardiac arrhythmias. Following initiation of memantine therapy, we observed an increase in the number of days during which the patient was more oriented and exhibited better short-term recall. On these days, her Folstein MMSE score fluctuated between 18-22/30. These fluctuations were primarily seen in the orientation portion of the MMSE. She frequently appeared less confused and less agitated during the evenings compared to her state prior to memantine therapy. We also observed an improvement in her late-day confusion. It is possible these changes are a result of improved nutrition with optimized antipsychotics and frequent reorientation over the duration of her lengthy admission. Additionally, it can propose a synergistic effect of quetiapine with memantine resulting in less agitation, which led to improved short-term memory. Family history with similar presentation in patient’s father elicits further evaluation for genetic (inherited) predisposition to Korsakoff syndrome, especially within subpopulations of chronic alcohol abuse and eating disorders, which have been largely considered multifactorial. Korsakoff syndrome is considered to be caused by long-term thiamine deficiency although not all populations with chronic, severe deficiencies progress to Korsakoff syndrome.

Korsakoff syndrome is hypothetically a result of glutamate excitotoxicity, and NMDA receptors are a class of glutamate receptors. An NMDA antagonist like memantine may prevent progression of disease and possibly even provide limited improvement in cognitive symptoms. The results we observed in our case are similar to those seen in a study performed in 2003 by Rustembeovic et al., in which 16 patients with Korsakoff syndrome were started on memantine. In the study, the test group showed significantly more positive outcomes in cognition and functional assessments compared to the placebo with less depreciation of ADLs [9]. With a small trial size, it is possible that those outcomes may be due to confounding factors. Another study by Cheon et al. showed similar improvement over a 12-week period with memantine therapy, however, they were unable to rule out the effect of prolonged alcohol cessation on patient improvement [10].

## Conclusions

We highlighted a single case that showed variable results with the use of memantine in addition to thiamine supplementation and cognitive therapy. Our objective in this report is to add to the limited body of data regarding treatment of Korsakoff syndrome and the use of memantine in said treatment. Further clinical studies with an increased number of participants are needed to determine the clinical efficacy and nootropic effect of memantine in Korsakoff syndrome patients.
